# Commentary: Current Practices and Trends of Plastic and Oncoplastic Breast Surgeons in Canada

**DOI:** 10.1177/22925503231224195

**Published:** 2024-01-02

**Authors:** Andrew Gorgy, Tyler Safran, Joshua Vorstenbosch

**Affiliations:** 1 Department of Plastic and Reconstructive Surgery, 54473 McGill University Health Center, Montreal, Canada

Oncoplastic breast surgeries combine oncological procedures with plastic surgical principles.^1^ Despite the significant benefits that oncoplastic surgery affords to patients, questions remain regarding current practices in Canada. In their cross-sectional survey of Canadian surgical oncologists and plastic surgeons, Bitoiu et al describe current oncological surgical practices as well as comfort levels of general and plastic surgeons with the various levels of reconstruction.^2^

This study demonstrates the delineation of responsibilities between breast surgeons and plastic surgeons in the execution of varying tiers of oncoplastic surgical procedures. The illumination of these divergences bears potential significance in safeguarding the role of plastic surgeons within the realm of oncoplastic surgeries. Like Clough et al, this paper makes a noteworthy contribution by validating previous findings about the categorization of level 1 procedure, which predominantly fall within the purview of breast surgeons, while reserving the more intricate level 2 and level 3 surgeries for plastic surgeons.^3^ Furthermore, this inquiry reveals a pivotal perspective, with all plastic surgeons expressing a high degree of competence in executing contralateral balancing procedures and advocating for their inclusion under the jurisdiction of plastic surgery, in contrast, breast surgeons contend that this responsibility can be managed by either a breast or a plastic surgeon. However, it is noteworthy that breast surgeons were the exclusive group to identify certain impediments in the execution of oncoplastic surgeries, including a lack of familiarity with specific techniques, the absence of dedicated billing codes, and protracted OR time.

Barriers to the adoption of oncoplastic surgical procedures, seen as impeding factors contributing to low rates of immediate reconstruction, were identified in this study. Breast surgeons highlight the lack of available plastic surgeons and insufficient formal oncoplastic training as major hindrances. Notably, many participating breast surgeons work in community practices, possibly influencing these findings. The study adeptly proposes solutions, such as formalizing oncoplastic training akin to European standards to enhance the competence of young breast surgeons. While acknowledging the time-consuming nature of implementing such policies in Canada, the authors recommend fostering collaboration between breast and plastic surgeons and establishing a referral network.^4^ This initiative holds promise for delivering timely care to patients with breast cancer, enhancing their quality of life, and sustaining plastic surgeons’ involvement in the oncoplastic domain.

On the other hand, it is important to discuss some of the limitations of this study that was already mentioned by the authors. For instance, the 61% response rates might introduce selection bias, especially that most surgeons who responded were in community practice. This could impact the generalizability of the results of the study. Moreover, we content that a more comprehensive exploration of the barriers faced by breast surgeons and potential solutions would be beneficial.

We congratulate the authors on their article and providing valuable insights into the perspectives of breast and plastic surgeons on oncoplastic surgery in Canada. Understanding these insights will come valuable in fostering the relationship between plastic and breast surgeons and will ensure the adequate delivery of care to patients with breast cancer.
